# Fortification of *Chlorella vulgaris* with citrus peel amino acid for improvement biomass and protein quality

**DOI:** 10.1016/j.btre.2023.e00806

**Published:** 2023-06-19

**Authors:** Zhila Heydari Koochi, Kourosh Ghodrat Jahromi, Gholamreza Kavoosi, Asghar Ramezanian

**Affiliations:** a Department of Biotechnology, School of Agriculture, Shiraz University, Shiraz, Iran; b Department of Horticultural Science, School of Agriculture, Shiraz University, Shiraz, Iran

**Keywords:** Citrus peel, *Chlorella vulgaris*, Biomass, Amino acid profile, Protein quality

## Abstract

The amino acid extract (protein hydrolysate) from various citrus peels was employed as an organic nutrition source for the culture of *Chlorella* to investigate their effects on the biomass and protein quality of the microalgae. The major amino acids in citrus peels included proline, asparagine, aspartate, alanine, serine, and arginine. The most plentiful amino acids in the *Chlorella* were alanine, glutamic acid, aspartic acid, glycine, serine, threonine, leucine, proline, lysine, and arginine. Adding the citrus peel amino acid extracts to the *Chlorella* medium enhanced overall microalgal biomass (more than two folds *p* < 0.05) and protein content (more than 1.25 fold, *p* < 0.05). Citrus peel amino acids increase essential amino acids and decrease the non-protein amino acid of *Chlorella* (*p* > 0.05). The present research shows that citrus peels have good nutritional quality and could be used for the inexpensive cultivation of *Chlorella* biomass with potential utility for food application.

## Highlight

• The major amino acids in citrus peels include proline, asparagine, aspartate, alanine, serine, and arginine.
• Adding the amino acid extracts to the *Chlorella* medium enhanced microalgal biomass and protein content.
• Citrus peel amino acids increase essential amino acids and decrease the non-protein amino acid of *Chlorella*.

## 1 Introduction

Microalgae are ideal for the food sector because of their fast growth rate and ability to survive in harsh conditions. There are several benefits to utilizing microalgae as a primary source of lipid and protein, including; fast growth, high productivity, the capacity to endure severe circumstances, the potential to be farmed using chemical waste, and the indirect reduction of greenhouse gas emissions [1]. Proteins, lipids, carbohydrates, vitamins, pigments, and carotenoids are all found in microalgae, and microalgae have been linked to favorable health outcomes such as decreased blood glucose, blood pressure, and cholesterol [2]. Microalgae are high in proteins, essential and non-essential amino acids, lipids, sugars, essential fatty acids, vitamin precursors, mineral deposits, organic acids, terpenoids, alkaloids, steroids, and phenolic compounds, all of which have been used as therapeutic foods to prevent hypertension, hypercholesterolemia, atherosclerosis, and diabetes mellitus [3]. A nutritious and affordable substance for microalgae feeding is required to employ *Chlorella* in the food and pharmacological industries and create bioethanol and biofuel. These objectives can be met by feeding microalgae with nutritive ingredients and promoting fatty acid production [4]. An inexpensive and nutritious material is required to feed *Chlorella* in the pharmaceutical and food sectors and encourage microalgae to synthesize proteins and amino acids [5].

Citrus is one of the most extensively grown horticultural fruits. The orange, lemon, lime, grapefruit, and mandarin are the most extensively farmed and industrially important fruits globally in the agro-industrial area. Juice for beverages, jellies, marmalades, potpourris, jams, candied peel, flavoring mediator for beverages, oils and essential oils, fiber, and pectin for food components are the main ingredients that are affordable in the citrus processing industry [6]. Citrus fruit is mostly made up of pulp, peel, and seeds. Pectin, citric acid, pentosans, fiber, glucoside, minerals, proteins, essential oils, and lipids make up the dry matter [7]. The most frequent derivative of the citrus processing industry is citrus peel waste. Peels, internal tissue, seeds, essential oil, and nutrients, for instance, amino acids and fatty acids, make up citrus peel waste [8]. Citrus peel waste is now utilized primarily as animal feedstuff, an organic soil conditioner, a composting substrate, and a bioethanol and biomethanol production substrate. Citrus peel waste has a high added value and may be used to make pectin, dietary fibers, protein, and fatty acids in the food sector. Citrus peel waste might potentially be used to extract flavonoids, favorable agents, and citric acid in the cosmetic and pharmaceutical sectors [9]. The macronutrient composition and profile of amino acids have been investigated to a lesser extent. Citrus peel waste with good nutritional quality offers enough nutrients for microalgae culture to enhance protein content in the microalgae. But the direct use of citrus peel has several limitations to microalgae culture due to antinutrient materials in the citrus peels. Partial extraction of amino acids and removing antinutrients could reduce these limitations [5].

Accordingly, in this research, the amino acid extract from different whole citrus peels was used as the organic nutrient source for cultivating *Chlorella*. The quality of extracted amino acids from bitter orange (*Citrus aurantium*), grapefruit (*Citrus paradisi*), sweet orange (*Citrus sinensis*), and mandarin (*Citrus reticulata*) peel waste and the stimulatory effects of their amino acid extracts on the manipulation of amino acid composition and protein quality of *Chlorella* are presented. The macronutrient composition, protein quality, and amino acid profile of citrus peel and *Chlorella* supplemented with amino acid extract from citrus peel have been explored.

## 2 Materials and methods

### 2.1 Plant materials and biochemical analysis

The bitter orange, sweet orange, grapefruit, and mandarin were grown at Fars Research Center for Agriculture and Natural Resources (Jahrom, Fars, Iran). Fresh peels from ripped and harvested fruits were obtained from randomly chosen and healthy trees in December. Jahrom Agricultural and Natural Resources Research Station are allocated at an altitude of 1070 m with a longitude of 53°, 37 ʹ, 33ʺ, and a latitude of 28°, 30ʹ, 48ʺ. Jahrom Agricultural and Natural Resources Research Station confirmed all the source and batch number details. Plant field studies and experimental research, including collecting plant material, are conducted following relevant institutional, national, and international guidelines and legislation. The peel portion (albedo and flavedo) was separated from the fruit and dried out in the shade before being crushed using a household grinder. Citrus peel waste was evaluated for moisture content, ash content, total carbohydrate, total protein, total lipid, protein digestibility, and total energy using standardized techniques provided in the literature by following the Association of Official Analytical standard procedures Chemist's method (AOAC) [4,10].

The chemical components of citrus peels and *Chlorella* supplemented with citrus peel were characterized using Fourier transforms infrared (FT-IR) spectroscopy in the range of 4000–400 cm^−1^, performed with a Bruker FTIR spectrophotometer (Germany).

### 2.2 Amino acid extraction and profiling

Plant materials (10 g) were mixed with 100 mL of 6 M HCl and incubated for one day at 100 °C. Cheesecloth was used to separate the plant components. The amino acid then powdered by lyophilization. For amino acid analysis and microalgae supplementation amino acid powder were solubilized in NaCl (0.75%). The amino acids were analyzed using liquid chromatography-mass spectrometry (LC-MS/MS) on an Agilent mass spectrometer (G1313). To eliminate particles from the amino acids profile, 2.0 mL of the amino acids extract was centrifuged at 3000 *×* g for 20 min. At a temperature of 40 °C and a 0.01 mL injection volume, the amino acids were separated using an amino acid analyzer column (C18 reversed-phase, length=100 mm, inner diameter=3 mm, pore size=100, particle size= 2.7 m). The mobile phase solutions were 0.2% formic acid in water (A) and 0.2% formic acid in acetonitrile (B). The elution protocol followed a linear gradient, starting at 5.0% solution B and increasing to 100% in 15 min. The flow rate of the mobile phase was 0.25 mL/min. The ion electrospray ionization (ESI) source obtained the mass spectrum in the *m/z* range of 30–3000 [11,12].

### 2.3 Protein quality

Total non-essential amino acids (NEAA), total essential amino acids (EAA), aromatic amino acids (AAA), non-protein amino acids (NPAA), hydrophobic amino acids (HAA), flavor amino acids (FAA), sulfur amino acids (SuAA), bitter amino acids (BAA), sweet amino acids (SwAA), protein efficiency ratio-1 (PER1), and protein efficiency ratio-2 (PER2) were calculated by using the formula described in the literature [13].

### 2.4 Microalgal culture and treatment

*Chlorella vulgaris* (IBRC: M50026) was obtained from the Iranian Biological Resources Center (IBRC) (Tehran, Iran). *Chlorella* was isolated using the agar plate procedure, then purified in Bold's Basal Media (BBM) before being transferred to the same liquid medium. The *Chlorella* cells were transferred to BBM liquid medium, bubbled, and maintained at 28 °C with an initial pH of 6.8 and 2500 E.m^−2^. s^−1^ light intensity to raise biomass. A mechanical pump aerated the culture after it passed through a 0.22 mm filter. The microalgal cells were supplied with amino acid (5.0 g/1000 mL) at the logarithmic phase, and the cultures were incubated for four days. The *Chlorella* biomass was collected at the end of the logarithmic phase by centrifugation, washed with deionized water to eliminate adhering residues, and dried by lyophilization [11,12]. Optical density (at 680 nm) and dry cell weight analyses were used to quantify cell proliferation [4]. Microalgae were evaluated for moisture content, ash content, total carbohydrate, total protein, total lipid, protein digestibility, and total energy using standardized techniques provided in the literature by following the Association of Official Analytical Standard Procedures Chemist's method (AOAC) [4,10].

### 2.5 Statistical analysis

The data is presented as a three-replicate average. Experimental data were evaluated using one-way analysis of variance (ANOVA) and Tukey post-hoc testing using SPSS software version 16 (SPSS Inc., Chicago, IL, USA). The matrix of amino acid composition data in the samples was analyzed for major components to investigate the apparent difference in the distribution of the components between the samples. Minitab (version 20.1.2) was used for the principal component analysis (PCA).

## 3 Results and discussion

### 3.1 Biochemical composition of citrus peel

**Fig. 1:**
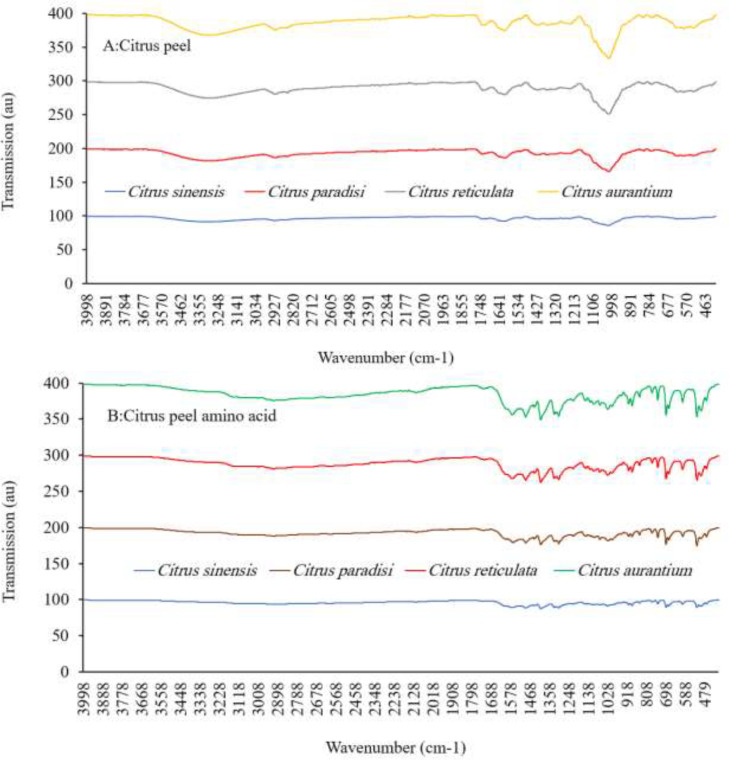
Fourier transforms infrared spectrum of citrus peel (A) and citrus peel amino acid (B) from *Citrus sinensis*, Citrus *paradisi, Citrus aurantium*, and *Citrus reticulata*.

The typical FTIR spectrum of bitter orange, sweet orange, grapefruit, or mandarin peels is shown in Fig. 1. The 3700–3100 cm^−1^ bands are associated with stretching vibrations of OH in water or hydrogen-bonded OH. Sharp peaks in the 2900–2700 cm^−1^ region are associated with the stretching vibrations of lipid and fatty acid CH, CH2, and CH3 groups. The stretching of C = O amides, C = C aromatics, N—H, or carboxyl in proteins is represented by the bands in the 1600–1300 cm^−1^ region. The bands at 1100–900cm^−1^ are caused by polysaccharide vibrations, including symmetric stretching of OH and C—O-C. The vibration of terpenes and terpenoids causes the bands at 900–500 cm^−1^. According to FTIR analyses, citrus peels had a high concentration of carbohydrates and a lower concentration of protein, fatty acids, and terpenes [14].

The macronutrient composition of bitter orange, sweet orange, grapefruit, and mandarin is summarized in Table 1. Carbohydrates (56.10–58.83 g / 100 g dry matter), proteins (17.61–19.20 g / 100 g dry matter), and lipids (11.56–13.83 g / 100 g dry matter) are the main macronutrients in the analyzed citrus peels. The macronutrient composition of bitter orange, sweet orange, grapefruit, and mandarin is similar, although statistically significant differences exist (*P* ≤ 5%). Carbohydrates are the major macronutrient in citrus peels, but the most abundant carbohydrates are fibers (pectin) that cannot be digested [15]. Bitter orange and grapefruit have the similar dry matter, carbohydrate, and fat content. Sweet orange and mandarin have similar protein, moisture, ash contents, and digestibility (Table 1 and Figure S1 in the supplemental file). As reported in the previous research, *Citrus maxima* [6] and *Citrus natsudaidai* [16] peels have similar chemical compositions to our findings. Our experimental results and previous work confirmed that citrus peel waste contains macronutrients (carbohydrates, proteins, lipids) and micronutrients (vitamins and minerals), and volatile constituents (polyphenols and flavonoids) that make citrus peels as the raw and inexpensive materials for foodstuff, food ingredients, and food flavoring [17,18].

**Table 1:** Biochemical composition of citrus peel from bitter orange (*Citrus aurantium*), grapefruit (*Citrus paradisi*), sweet orange (*Citrus sinensis*), and mandarin (*Citrus reticulata*).

Parameter	Bitter orange	Grapefruit	Sweet orange	Mandarin
Moisture (g/g fw)	77.50±2.77^a^	75.40±2.84^a^	78.52±2.74^a^	80.32±2.68^a^
Dry matter (g/g fw)	22.50±1.29^a^	24.60±1.33^a^	21.48±1.37^ab^	19.68c±1.33^b^
Ash (g/100 g dm)	8.54±0.97^a^	9.56±1.02^a^	10.85±1.12^a^	9.63±0.98^a^
Carbohydrate (g/100 g dm)	57.42±3.63^a^	58.83±3.60^a^	56.10±3.65^a^	57.85±3.66^a^
Protein (g/100 g dm)	18.50±1.47^a^	17.61±1.44^a^	18.99±1.43^a^	19.20±1.40^a^
Lipids (g/100 g dm)	13.83±1.69^a^	12.89±1.47^a^	12.67±1.21^a^	11.56±1.14^a^
Energy (kcal/100 g)	428.15±11.84^a^	421.78±12.80^a^	414.39±10.86^a^	412.24±9.86^a^
Digestibility (g/100 g)	53.70±3.30^a^	52.60±3.50^a^	54.33±3.45^a^	54..25±4.00^a^

The values are expressed as means ± SD for three replicates. Mean values with different letters within a row are significantly different by the Tukey test (*p* < 0.05).

### 3.2 Amino acid composition of citrus peel

**Fig. 2:**
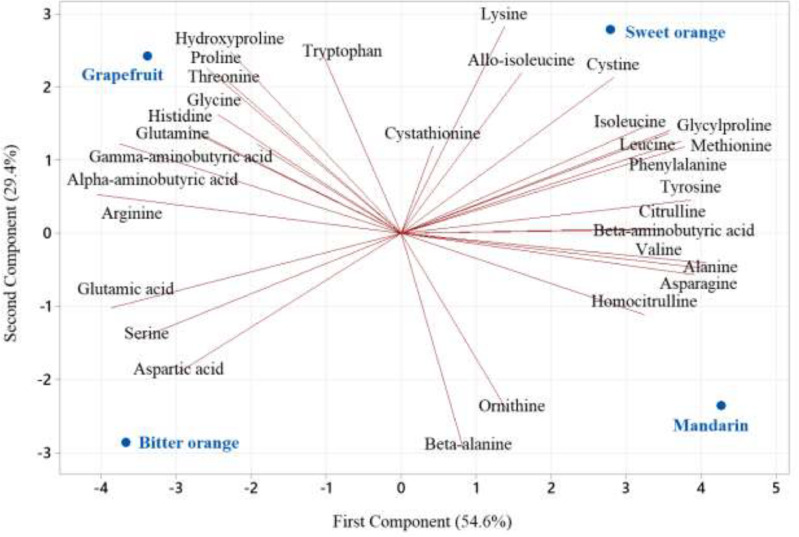
Principal Component Analysis (PCA) biplot illustrating the relationships among the amino acid composition of bitter orange (*Citrus aurantium*), grapefruit (*Citrus paradisi*), sweet orange (*Citrus sinensis*), and mandarin (*Citrus reticulata*).

Amino acids from citrus peels have a slightly similar FTIR spectrum, represented by bands in the 1700–500 cm^−1^ area (Fig. 1). The amino acid extract possesses a high concentration of amino acids and a low quantity of phenolic hydrocarbon and terpenes, according to FTIR analysis [14]. LC-MS/MS results show that proline, asparagine, aspartate, alanine, beta-aminoisobutyric acid, serine, arginine, gamma-aminobutyric acid, glutamate, glycine, and valine are the major amino acids found in citrus peel (Table 2). Mandarin and sweet orange had similar amounts of valine, alanine, asparagine, tyrosine, phenylalanine, leucine, isoleucine, methionine, citrulline, beta-aminobutyric acid, glycylproline, homocitruline, and cysteine (*P* ≤ 5%). Bitter orange and grapefruit had similar amounts of arginine, glutamic acid, alpha-aminobutyric acid, serine, aspartic acid, histidine, glutamine, proline, glycine, threonine, hydroxyproline, and gamma-aminobutyric acid (*P* ≤ 5%). The most abundant amino acids are leucine, threonine, lysine, phenylalanine, and histidine. Tryptophan and cysteine are the restricted amino acids (Table 2 and Fig. 2). *Citrus natsudaidai* peel [16] contains similar amounts of amino acids as found in this study.

**Table 2:** Amino acid composition (g/100 g protein) of citrus peel from bitter orange (*Citrus aurantium*), grapefruit (*Citrus paradisi*), sweet orange (*Citrus sinensis*), and mandarin (*Citrus reticulata*).

Amino acid	Bitter orange	Grapefruit	Sweet orange	Mandarin	WHO70
Alanine	5.797±0.32^c^	5.981±0.33^c^	8.473±0.47^b^	10.515±0.58^a^	0.000
Allo-isoleucine	0.002±0.00^a^	0.004±0.00^a^	0.013±0.00^a^	0.002±0.00^a^	0.000
Alpha-aminobutyric acid	0.058±0.00^a^	0.067±0.00^a^	0.045±0.00^a^	0.024±0.00^a^	0.000
Arginine	6.619±0.36^a^	7.502±0.41^a^	2.717±0.15^b^	0.751±0.04^c^	0.000
Asparagine	16.133±1.35^c^	17.718±1.27^c^	26.427±1.45^b^	36.851±2.03^a^	0.000
Aspartic acid	15.510±1.25^a^	7.746±0.43^c^	4.069±0.22^d^	6.258±0.34^c^	0.000
Beta-alanine	0.119±0.01^a^	0.106±0.01^a^	0.109±0.01^a^	0.120±0.01^a^	0.000
Beta-aminoisobutyric acid	6.726±0.57^b^	4.463±0.25^c^	10.336±0.75^a^	8.473±0.47^a^	0.000
Citrulline	0.034±0.00^a^	0.047±0.00^a^	0.051±0.00^a^	0.067±0.00^a^	0.000
Cystathionine	0.001±0.00^a^	0.008±0.00^a^	0.003±0.00^a^	0.006±0.00^a^	0.000
Cystine	0.009±0.00^a^	0.019±0.00^a^	0.033±0.00^a^	0.021±0.00^a^	0.420
Gamma-aminobutyric acid	4.098±0.23^a^	2.745±0.15^b^	4.495±0.25^a^	0.060±0.00^c^	0.000
Glutamic acid	2.081±0.41^a^	1.677±0.29^a^	0.735±0.04^b^	0.901±0.05^b^	0.000
Glutamine	1.403±0.08^b^	2.315±0.13^a^	1.236±0.07^a^	1.280±0.07^b^	0.000
Glycine	1.227±0.07^a^	1.620±0.09^a^	1.216±0.07^a^	1.196±0.07^a^	0.000
Glycylproline	0.008±0.00^a^	0.015±0.00^a^	0.056±0.00^a^	0.032±0.00^a^	0.000
Histidine	0.268±0.01^a^	0.538±0.03^a^	0.219±0.01^a^	0.201±0.01^a^	0.700
Homocitrulline	0.002±0.00^a^	0.004±0.00^a^	0.005±0.00^a^	0.017±0.00^a^	0.000
Homocysteine	0.002±0.00^a^	0.004±0.00^a^	0.001±0.00^a^	0.003±0.00^a^	0.000
Hydroxylysine	0.002±0.00^a^	0.004±0.00^a^	0.004±0.00^a^	0.001±0.00^a^	0.000
Hydroxyproline	0.065±0.00^b^	0.104±0.01^a^	0.088±0.00^b^	0.028±0.00^b^	0.000
Isoleucine	0.224±0.01^b^	0.299±0.02^b^	0.506±0.03^a^	0.413±0.02^a^	0.840
Leucine	0.272±0.01^b^	0.487±0.03^b^	0.971±0.05^a^	0.815±0.04^a^	1.750
Lysine	0.560±0.03^b^	1.027±0.06^a^	1.125±0.06^a^	0.804±0.04^b^	1.400
Methionine	0.119±0.01^b^	0.150±0.01^b^	0.264±0.01^a^	0.210±0.01^a^	0.700
Ornithine	0.822±0.05^b^	0.475±0.03^b^	0.198±0.01^c^	1.538±0.08^a^	0.000
Phenylalanine	0.354±0.02^b^	0.493±0.03^b^	0.706±0.04^a^	0.667±0.04^a^	1.050
Proline	25.643±1.41^b^	34.980±1.92^a^	27.737±1.53^b^	21.201±1.17^c^	0.000
Serine	8.888±0.89^a^	6.178±0.34^b^	4.195±0.23^c^	4.506±0.25^c^	0.000
Threonine	0.804±0.04^a^	0.877±0.05^a^	0.902±0.05^a^	0.483±0.03^a^	1.050
Tryptophan	0.076±0.00^b^	0.297±0.02^a^	0.174±0.01^a^	0.116±0.01^a^	0.280
Tyrosine	0.217±0.01^d^	0.424±0.02^c^	0.620±0.03^b^	0.769±0.04^a^	0.700
Valine	0.868±0.05^b^	0.743±0.04^b^	1.392±0.08^a^	1.566±0.09^a^	1.260
Total	99.010±3.45^a^	99.116±4.75^a^	99.120±5.5^a^	99.897±4.49^a^	10.150
Non-essential amino acid	83.527±4.59^a^	86.159±4.74^a^	77.457±4.26^a^	84.250±4.63^a^	1.120
Essential amino acid	3.544±0.19^b^	4.910±0.27^ab^	6.259±0.34^a^	5.277±0.29^a^	9.030
Non-protein amino acid	11.939±1.16^b^	8.047±0.44^c^	15.404±1.85^a^	10.370±0.87^b^	0.000
Flavor amino acid	17.591±1.27^a^	9.423±0.52^b^	4.804±0.26^d^	7.159±0.39^c^	0.000
Aromatic amino acid	0.647±0.04^b^	1.214±0.07^a^	1.500±0.08^a^	1.553±0.09^a^	2.030
Sweet amino acid	42.359±2.33^b^	49.635±2.73^a^	42.523±2.34^b^	37.902±2.08^c^	1.050
Bitter amino acid	7.931±0.44^b^	9.766±0.54^a^	5.557±0.31^c^	3.174±0.17^d^	5.320
Sulfur amino acid	0.128±0.01^a^	0.169±0.01^a^	0.297±0.02^a^	0.231±0.01^a^	1.120
Hydrophobic amino acid	33.276±1.83^c^	43.132±2.37^a^	40.049±2.20^ab^	35.388±1.95^c^	5.600
Protein efficiency ratio-1	−1.765±−0.10^a^	−2.106±−0.12^a^	−1.545±−0.08^a^	−1.309±−0.07^a^	0.114
Protein efficiency ratio-2	−0.367±−0.02^a^	−0.292±−0.02^a^	−0.092±−0.01^b^	−0.179±−0.01^a^	0.253

The values are expressed as means ± SD for three replicates. Mean values with different letters within a row are significantly different by the Tukey test (*p* < 0.05).

### 3.3 Amino acid nutritional quality of citrus peel

Table 2 summarizes the protein quality of citrus peel in terms of amino acids. Sweet orange and mandarin are similar according to PER2, SuAA, EAA, AAA, PER1, and NPAA (*P* ≤ 5%). Grapefruit and bitter orange have the same amounts of FAA, NEAA, BAA, and SWAA (*P* ≤ 5%) (Table 2 and Figure S2 in supplemental file). Citrus peels have a low EAA content; however, compared to the amino acid necessities of adults, they can be a good supply of EAA [16]. NPAA in citrus peels, such as aminobutyric acid, ornithine, and citrulline, are critical for plant physiology and development, carbon/nitrogen balance, energy dissipation, fruit growth, and immune response enhancement [19]. FAA produces umami flavors related to the palatability of food and its beneficial effects in regulating gastrointestinal function and treating clinical gastrointestinal diseases and stomach diseases [20]. SAA and BAA can make meals taste more natural. The amount of tasty amino acids found in citrus peel is much lower than the taste threshold. As a result, citrus peel amino acid does not affect the taste of food ingredients. Due to a lack of supply, SuAA has become the limited amino acid in plant proteins. SuAAs are involved in the formation of cellular antioxidants like n-acetylcysteine and glutathione. SuAA can also be used as a heavy metal chelating antidote to eliminate harmful metals from the body and prevent the accumulation of hazardous metal ions [21]. In addition, citrus peel usually contains other macronutrients (carbohydrates and lipids), micronutrients, natural antioxidants, and carotenoids, making it suitable for use as certain food additives.

### 3.4 Biochemical composition of *Chlorella vulgaris*

**Fig. 3:**
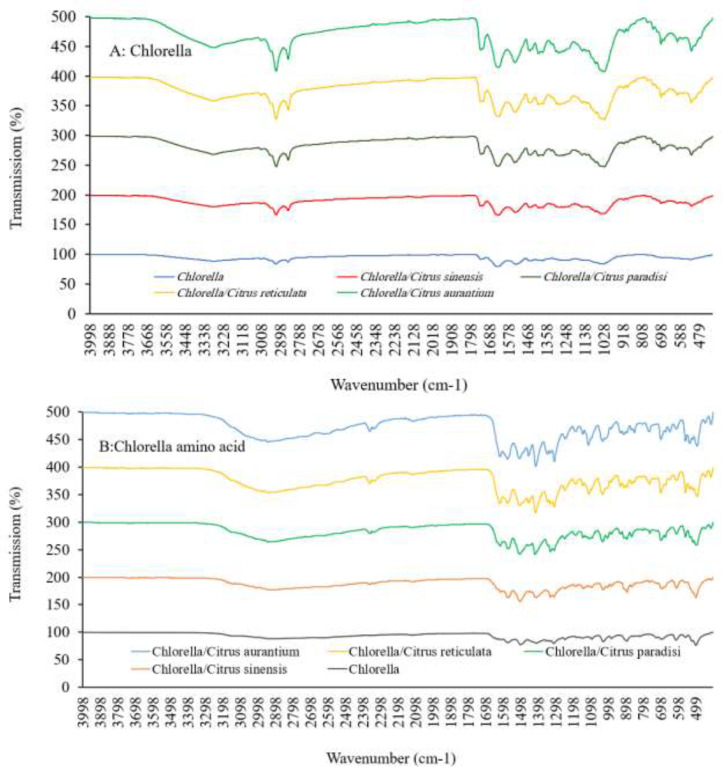
Fourier transform infrared spectrum of *Chlorella* (A) and *Chlorella* amino acid (B) supplemented with *Citrus sinensis, Citrus paradisi, Citrus reticulata,* and *Citrus aurantium* amino acid.

The FTIR patterns of *Chlorella* supplemented with citrus peels differ, indicating the various components in these products (Fig. 3). The 3700–3100 cm^−1^ region is associated with the stretching vibrations of OH in water and hydrogen bonds. The peaks in the 2900–2700 cm^−1^ region could be linked to the stretching vibration of lipid and fatty acids CH_2_, CH, and CH3. The majority of the variations in FTIR patterns observed in the 2900–2700 cm^−1^ region can be attributed to a variation in fatty acid composition. The C=C stretching of unsaturated fatty acids and the formation of MUFA and PUFA in *Chlorella* after supplementing with citrus peel can be related to the variations in FTIR patterns observed in the 1900–1500 cm^−1^. The bands in the 1300–1000 cm^−1^ range represent the stretching of C=O amides, C=C aromatics, N-H, and carboxyl groups in proteins. Polysaccharide vibrations, comprising symmetric stretching of the C—O-C and OH groups, produce the bands at 1100–900 cm^−1^. The vibration of terpenes and terpenoids causes the bands at 900–500 cm^−1^. FTIR findings suggest that *Chlorella* includes mostly proteins, carbohydrates, fatty acids, and lower levels of hydrocarbon and terpenes [22].

**Fig. 4:**
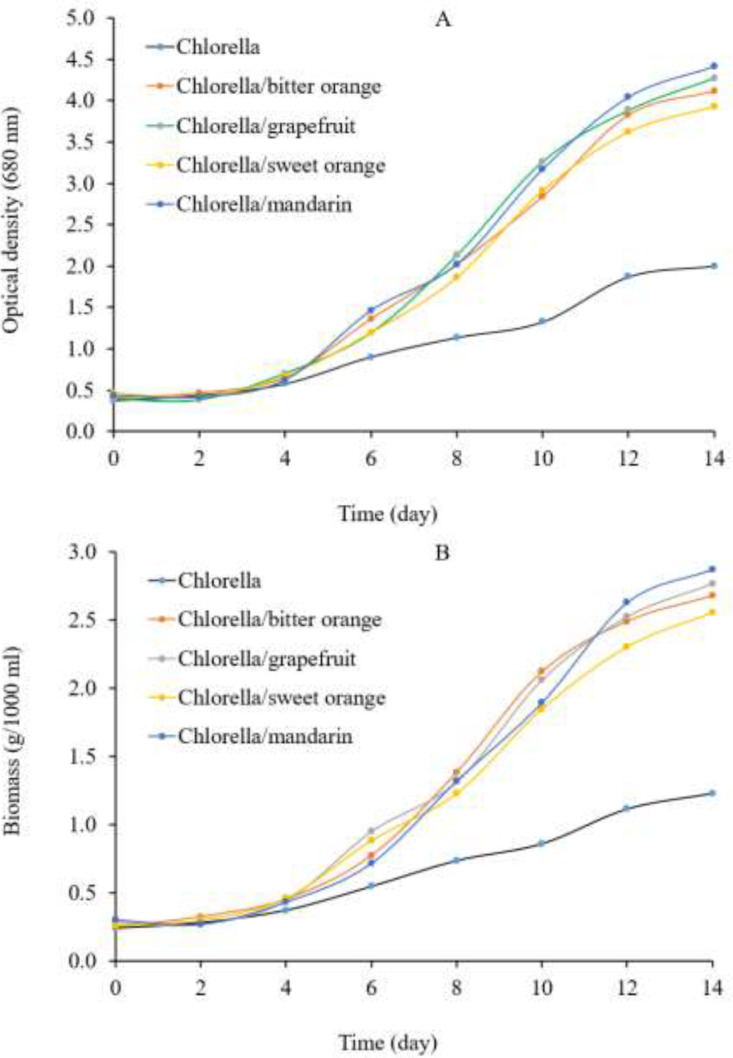
Optical density (A) and biomass (B) of Chlorella in the presence of protein hydrolysate (amino acids) from bitter orange (*Citrus aurantium*), grapefruit (*Citrus paradisi*), sweet orange (*Citrus sinensis*), and mandarin (*Citrus reticulata*).

For 14 days, optical density measurements and changes in dry mass levels were used to monitor *Chlorella* growth in the presence and absence of citrus peel protein hydrolysates (Fig. 4). *Chlorella* grows much faster in the citrus peel protein hydrolysates (amino acid) until 12 days reach to stationary phase. *Chlorella* supplementation with citrus peel amino acids increases total biomass (more than two folds *p* < 0.05) and protein content (more than 1.25 folds *p* < 0.05) while reducing carbohydrates (*p* < 0.05) and lipid (*p* < 0.05) to some extent (Table 3). Previous research also reported that protein hydrolysates and nitrate (amino acid as nitrogen source) could increase biomass and protein production in microalgae [4,12].

**Table 3:** Proximate analysis of *Chlorella vulgaris* supplemented with amino acid from citrus peel from bitter orange (*Citrus aurantium*), grapefruit (*Citrus paradisi*), sweet orange (*Citrus sinensis*), and mandarin (*Citrus reticulata*).

Parameter	Chlorella	Chlorella/bitter orange	Chlorella/grapefruit	Chlorella/sweet orange	Chlorella/mandarin
Biomass (g/1000 ml)	1.15±0.034c	2.65±0.065b	2.77±0.080a	2.53±0.076b	2.84±0.085a
Moisture (g/100 g fw)	6.75±0.64^a^	7.37±0.86^a^	6.86±0.74^a^	7.57±0.67^a^	7.45±0.56^a^
Dry matter (g/100 g fw)	93.25±3.32^a^	92.63±3.29^a^	93.14±3.31^a^	92.43±3.29^a^	92.55±3.29^a^
Ash (g/100 g dm)	7.85±0.58^a^	6.54±0.43^a^	6.65±0.54^a^	6.85±0.46^a^	6.63±0.50^a^
Sugar (g/100 g dm)	28.35±1.21^a^	23.32±1.53^b^	22.75±1.70^b^	23.81±1.85^b^	24.55±1.87^b^
Protein (g/100 g dm)	38.85±2.38^b^	47.50±2.69^a^	48.75±2.73^a^	48.99±2.74^a^	49.72±2.77^a^
Fat (g/100 g dm)	24.50±1.87^a^	21.83±1.78^ab^	21.20±1.75^ab^	19.67±1.70^b^	18.56±1.66^b^
Energy (kcal/100 g)	489.30±17.39^a^	479.75±17.06^a^	476.81±16.95^a^	468.23±16.65^a^	464.12±16.50^a^
Digestibility (g/100 g)	55.50±1.97^a^	57.73±2.05^a^	55.37±1.97^a^	56.33±2.00^a^	56.25±2.00^a^

The values are expressed as means ± SD for three replicates. Mean values with different letters within a row are significantly different by the Tukey test (*p* < 0.05). fw=fresh weight. dm=dry matter.

The macronutrient composition of *Chlorella* and *Chlorella* supplemented with citrus peel amino acids is stated in Table 3. Proteins (38.85 g / 100 g dry matter), carbohydrates (28.35 g / 100 g dry matter), and lipids (24.50 g / 100 g dry matter) are the main macronutrients in the *Chlorella* (Table 3). The previously reported *Chlorella* biomass contains an average of 40 gs of protein, 12 gs of fiber, 18 gs of carbohydrates, and 10 gs of lipids per 100 gs of dry biomass, to some extent differing from our experimental results, especially in lipids and carbohydrates [3,13]. Proteins (47.50–49.72 g / 100 g dry matter), carbohydrates (22.75–24.55 g / 100 g dry matter), and lipids (18.56–21.83 g / 100 g dry matter) are the main macronutrients in the *Chlorella* supplemented with different citrus peels protein hydrolysate (amino acid). *Chlorella* and *Chlorella*/grapefruit samples are closely similar according to fat, ash, energy, dry matter, and carbohydrate (*p* < 0.05). On the other hand, *Chlorella*/bitter orange, *Chlorella*/mandarin, and *Chlorella*/sweet orange are similar according to protein, moisture, and digestibility (*p* < 0.05) (Table 3 and Figure S3 in supplemental file). The carbohydrates (mainly starch) and dietary fiber (mainly cellulose) in the microalgae biomass could be possible raw materials for bioethanol synthesis and have increased interest due to their low concentration of lignin and hemicellulose. Compared to proteins and carbohydrates, the lipid content of *Chlorella* is relatively low because the algae culture is not affected by nutrients and other mechanisms to stimulate lipid production [3,4].

### 3.5 Amino acid composition of *Chlorella vulgaris*

Amino acids from *Chlorella* and supplemented *Chlorella* have a slightly comparable FTIR spectrum, mainly indicated by bands in the 1700–500 cm^−1^ (Fig. 3). The amino acid extract included a high concentration of amino acids and a small proportion of phenolic hydrocarbon and terpenes, according to FTIR studies [22]. Table 4 shows the amino acid composition of *Chlorella* supplemented with amino acids from bitter orange, grapefruit, sweet orange, and mandarin peel. The main amino acid components in *Chlorella* protein were alanine, serine, aspartic acid, glutamic acid, glycine, leucine, threonine, phenylalanine, proline, lysine, arginine, isoleucine, valine, tyrosine, citrulline, methionine, histidine, and aspartic acid, to some extent similar to other results [3,23]. Because there are some statistically significant alterations, the amino acid composition of *Chlorella* remains essentially constant after supplementation with citrus peel amino acids (*p* < 0.05). The amino acid profile of *Chlorella* and supplemented *Chlorella* are similar, but the total amino acids increased.

**Table 4:** Amino acid composition (mg/100 g protein) of *Chlorella vulgaris* supplemented with amino acid from citrus peel from bitter orange (*Citrus aurantium*), grapefruit (*Citrus paradisi*), sweet orange (*Citrus sinensis*), and mandarin (*Citrus reticulata*).

Amino acid	Chlorella	Chlorella/sweet orange	Chlorella/mandarin	Chlorella/grapefruit	Chlorella/bitter orange	WHO70
Alanine	15.53±1.89^a^	14.33±1.82^a^	15.00±1.85^a^	13.89±1.79^a^	14.16±1.81^a^	0.00
Alpha-aminobutyric acid	0.01±0.00^a^	0.02±0.00^a^	0.01±0.00^a^	0.02±0.00^a^	0.02±0.00^a^	0.00
Arginine	3.52±0.20^a^	3.38±0.19^a^	3.50±0.20^a^	3.11±0.18^a^	3.01±0.17^a^	0.00
Asparagine	1.01±0.06^a^	0.51±0.03^b^	0.57±0.03^b^	0.63±0.04^b^	0.60±0.03^b^	0.00
Aspartic acid	9.54±0.54^a^	8.93±0.51^a^	8.13±0.46^a^	8.04±0.46^a^	7.90±0.45^a^	0.00
Beta-alanine	1.24±0.07^a^	0.02±0.00^b^	0.02±0.00^b^	0.02±0.00^b^	0.03±0.00^b^	0.00
Beta-aminoisobutyric acid	0.09±0.01^b^	0.09±0.00^b^	0.07±0.00^b^	0.15±0.01^a^	0.16±0.01^a^	0.00
Citrulline	1.64±0.09^a^	0.49±0.03^b^	0.50±0.03^b^	0.57±0.03^b^	0.52±0.03^b^	0.00
Cysteine	0.09±0.01^a^	0.03±0.00^a^	0.04±0.00^a^	0.09±0.01^a^	0.10±0.01^a^	0.42
Gamma-aminobutyric acid	0.57±0.03^a^	0.33±0.02^a^	0.41±0.02^a^	0.43±0.02^a^	0.37±0.02^a^	0.00
Glutamic acid	9.83±0.56^a^	9.61±0.55^a^	9.59±0.55^a^	9.05±0.52^a^	9.21±0.52^a^	0.00
Glutamine	0.95±0.05^a^	0.26±0.02^b^	0.39±0.02^b^	0.44±0.02^b^	0.33±0.02^b^	0.00
Glycine	9.33±0.83^a^	11.22±1.64^a^	10.77±1.21^a^	11.24±1.40^a^	11.22±10.23^a^	0.00
Glycylproline	0.32±0.02^a^	0.07±0.00^b^	0.08±0.00^b^	0.08±0.00^b^	0.09±0.01^b^	0.00
Histidine	1.32±0.08^b^	1.87±0.11^a^	1.84±0.10^a^	1.69±0.10^a^	1.84±0.10^a^	0.70
Hydroxyproline	0.12±0.01^a^	0.11±0.01^a^	0.08±0.00^a^	0.11±0.01^a^	0.10±0.01^a^	0.00
Isoleucine	2.45±0.14^a^	2.40±0.14^a^	2.26±0.13^a^	2.15±0.12^a^	2.38±0.14^a^	0.84
Leucine	7.55±0.73^a^	7.65±0.64^a^	7.55±0.83^a^	7.47±0.63^a^	8.17±0.87^a^	1.75
Lysine	4.64±0.26^b^	6.39±0.36^a^	5.92±0.34^a^	6.98±0.40^a^	6.12±0.35^a^	1.40
Methionine	1.40±0.08^a^	1.21±0.07^a^	1.25±0.07^a^	1.37±0.08^b^	1.43±0.08^a^	0.70
Ornithine	0.55±0.03^a^	0.54±0.03^a^	0.75±0.04^a^	0.62±0.04^a^	0.59±0.03^a^	0.00
Phenylalanine	3.39±0.19^a^	3.77±0.21^a^	3.64±0.21^a^	3.70±0.21^a^	3.91±0.22^a^	1.05
Proline	5.11±0.29^b^	6.45±0.37^a^	6.74±0.38^a^	7.25±0.41^a^	7.26±0.41^a^	0.00
Serine	7.61±0.93^a^	8.44±0.88^a^	7.95±0.75^a^	8.37±0.78^a^	8.17±0.97^a^	0.00
Threonine	6.12±0.35^a^	6.63±0.38^a^	6.28±0.36^a^	6.67±0.38^a^	6.28±0.36^a^	1.05
Tryptophan	0.01±0.00^a^	0.01±0.00^a^	0.01±0.00^a^	0.02±0.00^a^	0.02±0.00^a^	0.28
Tyrosine	2.24±0.93^a^	1.97±0.71^a^	1.87±0.91^a^	2.11±0.52^a^	2.00±0.61^a^	0.70
Valine	3.26±0.79^a^	3.17±0.78^a^	3.35±0.89^a^	3.13±0.88^a^	3.51±0.92^a^	1.26
Total	99.46±5.67^a^	99.92±5.70^a^	98.55±5.62^a^	99.39±5.67^a^	99.50±5.67^a^	10.15
Non-essential amino acid	64.76±3.69^a^	65.15±3.71^a^	64.54±3.68^a^	64.22±3.66^a^	63.95±3.65^a^	1.12
Essential amino acid	30.14±2.72^a^	33.10±2.89^a^	32.09±2.83^a^	33.18±2.89^a^	33.65±2.92^a^	9.03
Non-protein amino acid	4.56±0.26^a^	1.67±0.10^b^	1.92±0.11^b^	1.99±0.11^b^	1.90±0.11^b^	0.00
Flavor amino acid	19.37±1.10^ab^	18.55±1.06^b^	17.72±1.01^b^	17.09±0.97^b^	17.11±0.98^b^	0.00
Aromatic amino acid	5.64±0.32^a^	5.75±0.33^a^	5.52±0.31^a^	5.83±0.33^a^	5.92±0.34^a^	2.03
Sweet amino acid	43.71±2.49^a^	47.07±2.68^a^	46.73±2.66^a^	47.42±2.70^a^	47.09±2.68^a^	1.05
Bitter amino acid	19.63±1.12^a^	20.29±1.16^a^	20.05±1.14^a^	19.50±1.11^a^	20.75±1.18^a^	5.32
Sulfur amino acid	1.49±0.08^a^	1.24±0.07^a^	1.29±0.07^a^	1.46±0.08^a^	1.52±0.09^a^	1.12
Hydrophobic amino acid	38.69±2.21^a^	38.98±2.22^a^	39.78±2.27^a^	38.96±2.22^a^	40.81±2.33^a^	5.60
Protein efficiency ratio-1	2.52±0.34^a^	2.50±0.24^a^	2.44±0.44^a^	2.38±0.54^a^	2.70±0.25^a^	0.11
Protein efficiency ratio-2	2.72±0.16^a^	2.80±0.16^a^	2.76±0.16^a^	2.70±0.15^a^	3.03±0.17^a^	0.25

The values are expressed as means ± SD for three replicates. Mean values with different letters within a row are significantly different by the Tukey test (*p* < 0.05).

**Fig. 5:**
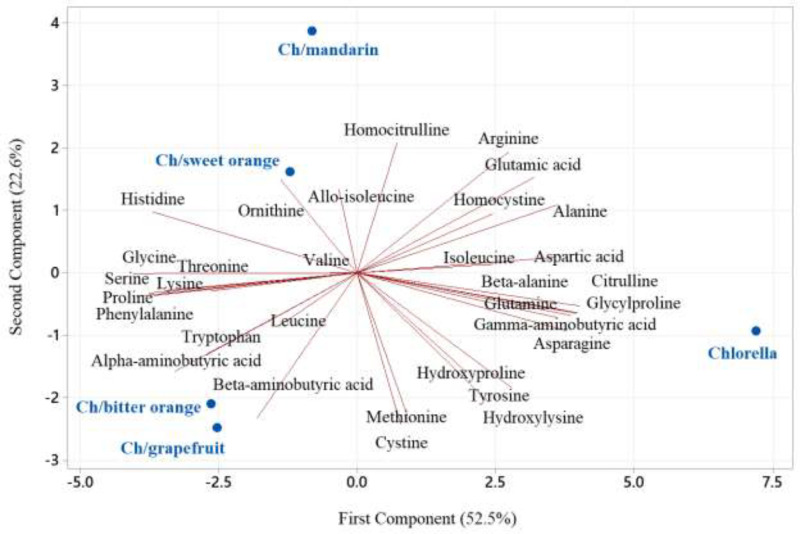
Principal Component Analysis (PCA) biplot illustrating the relationships among the amino acids composition of Chlorella vulgaris (Ch) supplemented with bitter orange (*Citrus aurantium*), grapefruit (*Citrus paradisi*), sweet orange (*Citrus sinensis*), and mandarin (*Citrus reticulata*).

*Chlorella* separated from *Chlorella* supplemented with citrus peel amino acids according to beta-alanine, citrulline, glycylproline, glutamine, asparagine, alanine, gamma-aminobutyric acid, aspartic acid, glutamic acid, tyrosine, arginine, homocysteine, isoleucine, hydroxylysine, and hydroxyproline (*p* < 0.05) (Table 4 and Fig. 5). *Chlorella*/bitter orange, *Chlorella*/grapefruit, *Chlorella*/sweet orange, and *Chlorella*/mandarin had similar amounts of glycine, proline, lysine, histidine, phenylalanine, serine, alpha-aminobutyric acid, tryptophan, threonine, beta-aminobutyric acid, and ornithine (*p* < 0.05) (Table 4 and Fig. 5). Threonine, leucine, lysine, phenylalanine, and histidine were the most common essential amino acids, while tryptophan and cysteine are the restricted amino acids (Table 4 and Fig. 5).

Liang et al. [24] reported lipid accumulation in *Chlamydomonas reinhardtii* by branched-chain amino acid catabolism through an increase in the acetyl CoA level. The amino acids could influence the contents of ω−6 and ω−3 fatty acids in *C. vulgaris* culture and improve the quality of microalgal fatty acids as a food supplement [25]. No comprehensive study has so far been reported on the techniques for promoting microalgal amino acid productivity by supplementing their culture media with citrus peel amino acids. Citrus peel amino acids can easily enter the catalytic pathway of the amino acid in microalgae and affect their levels. Amino acids are broken down into precursors or mediators of the citric acid cycle, most of which occur in the mitochondria (Figure S5 in the supplemental file). Citrus peel amino acids can enter the catabolism pathway of *Chlorella* through the glycine or any other amino acid and affects biomass, amino acid profile, and nutritional quality of microalgae [12]. Generally, adding amino acids as carbon and nitrogen sources resulted in better growth and increased total amino acid production. In general, the main amino acids of the citrus peels were proline, serine, aspartic acid, glycine, alanine, ornithine, glutamic acid, lysine, and valine, most of which are glucogenic. Ketogenic amino acids (leucine, lysine, tryptophan, phenylalanine, and tyrosine) are metabolized to acetyl CoA. Glucogenic amino acids (glutamic acid, glutamine, aspartic acid, asparagine, arginine, histidine, proline, valine, methionine, and threonine) are metabolized to glucose precursor and glucose metabolism pathway intermediates. Glucose is metabolized to acetyl-CoA by the glycolysis pathway. Acetyl-CoA produced in the mitochondria is the main building block for ATP production and synthesizing other amino acids under high-energy conditions. Acetyl-CoA produced from the catabolism of branched-chain amino acids can be used for the biosynthesis of some amino acids, causing an increase in amino acid accumulation [26].

Neutral non-polar and acidic polar amino acids are catabolized via extracellular amino acid oxidases. At the same time, the basic polar amino acids are transported to the cell. Inside the cell, amino acids are converted to nitrogen supply and alpha ketoacids by the activity of enzymes amino acid oxidase and dehydrogenase. The carbon skeletons of amino acids are generally converted to precursors or intermediates of the tricarboxylic acid cycle, which can contribute to ATP production and amino acid synthesis. Intermediates of the tricarboxylic acid cycle, including α-ketoglutarate, can convert to glutamic acid, the precursor for proline and arginine synthesis [26]. Succinyl-CoA and fumarate are precursors for synthesizing threonine, methionine, valine, isoleucine, tyrosine, and phenylalanine. Oxaloacetate can be converted to aspartic acid, asparagine, lysine, methionine, and threonine. Pyruvate and phosphoenolpyruvate can also be converted to valine, leucine, alanine, tyrosine, and phenylalanine. Aspartic acid converted to oxaloacetic acid, entered the tricarboxylic acid cycle, and produced energy and acetyl-CoA. Under amino acid supplementations, aspartic acid is transferred to the cytosol by the malate/aspartate shuttle and converted to threonine, methionine, and lysine. As can be seen, the levels of these amino acids have increased in *Chlorella* supplemented with citrus peel amino acids [26].

### 3.6 Amino acid nutritional quality of *Chlorella vulgaris*

Table 4 summarizes the amino acid nutritional quality of *Chlorella* cultured in the presence of amino acids from bitter orange, grapefruit, sweet orange, and mandarin. *Chlorella*/bitter orange, *Chlorella*/grapefruit, and *Chlorella/*sweet orange were similar according to EAA, SwAA, HAA, PER2, AAA, BAA, and PER1. While, *Chlorella* and *Chlorella/*mandarin were similar according to FAA, NPAA, and NEAA (Table 4, Figure S4 in supplemental file). Due to its high EAAs, and high biological value, *Chlorella* is more popular than bacteria and fungi as a single-cell protein source for human feeding. Microalgae have a greater protein nutritional quality than conventional plants. Other nutritional components in microalgae include carbohydrates, polysaccharides, lipids, vitamins, and minerals, which can be used as functional foods, supplements, and nutrients. Compared to WHO/FAO recommendations for adults and newborns, *Chlorella* may be an excellent source of EAA. *Chlorella* can improve the amino acid quality of foodstuffs as a food additive. Based on the interaction between proline-leucine (PER1) and tyrosine-leucine (PER2), the protein efficiency ratio (PER) evaluates the nutritional quality of food protein. Low-quality protein has a PER value of less than 1.5, whereas high-quality protein has a PER value of more than 2.0. The PER values for *Chlorella* are greater than 2, indicating that the protein from this microalga is of good quality [20,27].

Because of population growth and a health-conscious society concerned with overconsumption of fats and carbohydrates, dietary protein intake is rising. Agriculture, aquaculture, and the food industry have been working actively in recent years to increase protein product output from both production and processing aspects. Dietary proteins derived from animal sources are of the highest quality, containing well-balanced profiles of essential amino acids that generally exceed those of other food sources. However, due to low production efficiency and significant environmental impacts, together with the negative health impacts associated with the dietary intake of some animal products, the consumption of animal proteins has been declining over the past few decades. Researchers and product development specialists have been working closely to discover new protein sources, such as microalga-based ingredients. Microalgae have been recognized as strategic crops. Due to their vast biological diversity, they have distinctive phenotypic traits and interactions with the environment in the production of biomass and protein, offering possibilities for the production of large quantities of microalgal protein through manipulating growing systems and conditions and bioengineering technologies. Several microalgal species are currently exploited for various biological and industrial applications, including human foods, functional ingredients, cosmeceuticals, pharmaceuticals, animal and aquaculture feeds, fatty acids, alginates, carotenoids, wastewater treatment, and biofuels. Despite this, microalgae remain underexploited crops, and research into their nutritional values and health benefits is in its infancy. Only a small handful of microalgal species are being produced at a commercial scale for use as human food or protein supplements [13,27]. As our experimental results show, manipulating *Chlorella* with amino acids from citrus peel increases total biomass and protein content.

## 4 Conclusion

Citrus peel is rich in carbohydrates, proteins, and lipids. Given amino acid content, citrus peels are rich sours of proline, asparagine, aspartate, alanine, beta-aminoisobutyric acid, serine, arginine, gamma-aminobutyric acid, glutamine, glutamic acid, glycine, essential amino acid, and non-essential oil with good nutritional quality. With this nutritional quality, citrus peels could be used as an inexpensive nutrient for microalgae growth to improve microalgae biomass. But the direct use of citrus peel has several limitations due to antinutrient materials in the citrus peels. Partial extraction of amino acids and removing antinutrients could reduce these limitations. Supplementing *Chlorella* with amino acids from citrus peel increases total biomass and protein content. Although further research is needed to increase *Chlorella*'s protein and amino acid content, recent findings suggest that citrus peel may be utilized to grow microalgae and provide microalgal biomass for nutritional supplements at a low cost.

## Author contributions

Gholamreza Kavoosi helped to conceptualize and design the study. Kourosh Ghodrat Jahromi and Zhila Heydari Koochi were responsible for material preparation, data collecting, and analysis. Zhila Heydari Koochi wrote the original draft of the work. The draft manuscript was reviewed and edited by Gholamreza Kavoosi and Asghar Ramezanian. Gholamreza Kavoosi was involved in the final manuscript's funding, validation, supervision, review, and submission. The final manuscript has been read and approved by all writers.

## Funding

The fund for this research was provided by 10.13039/501100005071Shiraz University (grant No. 88-GR-AGRST-108).

## Availability of data and materials

On reasonable request, the corresponding author can provide the data supporting this study's findings.

## Ethics approval

In our research, no human subjects or animal studies were used.

## Declaration of Competing Interest

The authors declare that they have no known competing financial interests or personal relationships that could have appeared to influence the work reported in this paper.

## Appendix Supplementary materials

Image, application 1
