# Supplementary material for: Fortification of Chlorella vulgaris with citrus peel amino acid for improvement biomass and protein quality
Source: Biotechnol Rep (Amst). 2023 Jun 19;39:e00806. doi: 10.1016/j.btre.2023.e00806 (PMC10319642; doi:10.1016/j.btre.2023.e00806)
Supplement: Supplementary file 1 [file mmc1.doc]

**Fortification of *Chlorella vulgaris* with citrus peel amino acid for improvement biomass and protein quality**

Zhila Heydari Koochi^1^, Kourosh Ghodrat Jahromi^1^, Gholamreza Kavoosi^1*^, Asghar Ramezanian^2^

1. Department of Biotechnology, School of Agriculture, Shiraz University, Shiraz, Islamic Republic of Iran.

2. Department of Horticultural Science, School of Agriculture, Shiraz University, Shiraz, Islamic Republic of Iran.

*Corresponding author: ghkavoosi@shirazu.ac.ir

Running title: Nutritional quality of *Chlorella vulgaris*


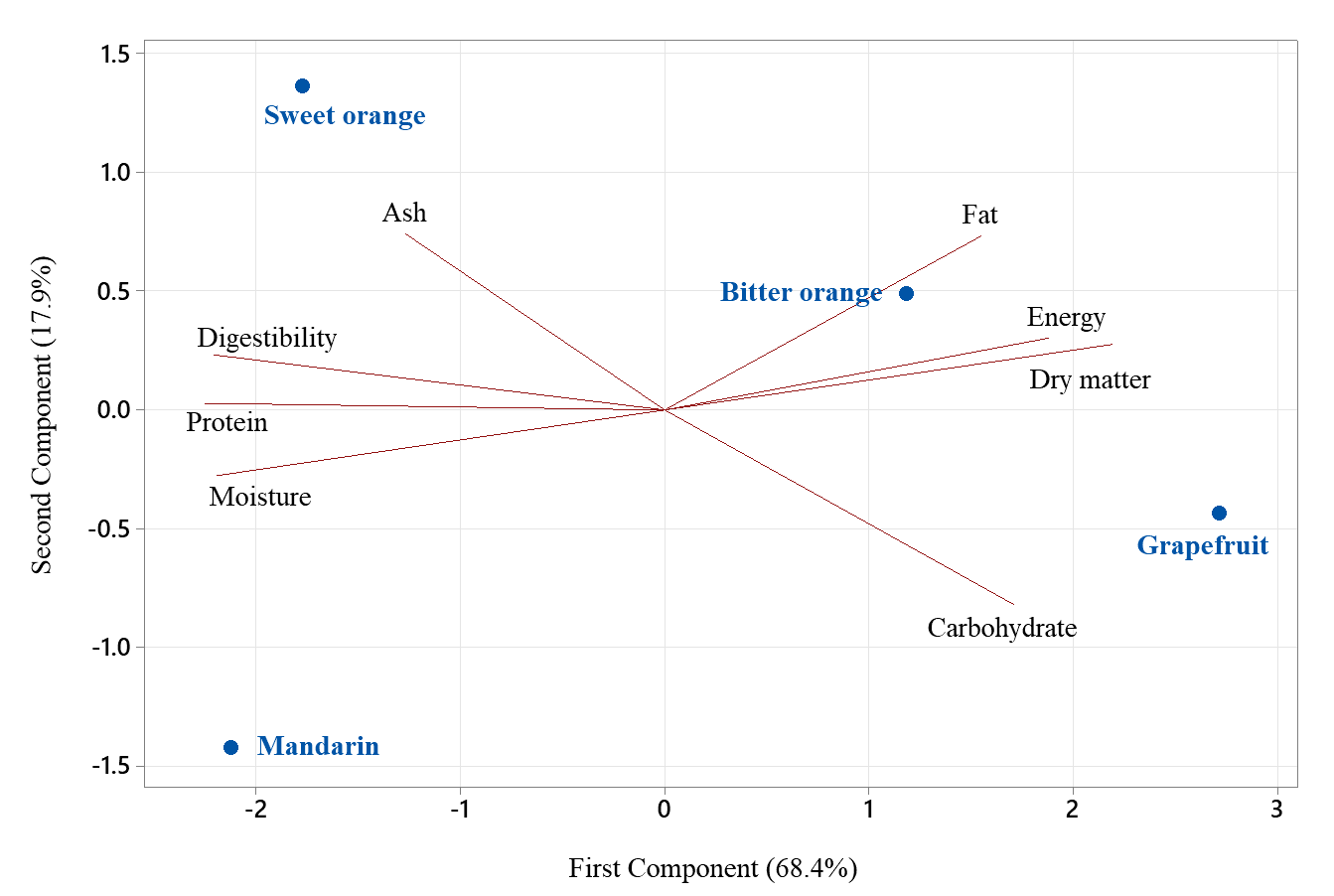


Figure S1. Principal Component Analysis (PCA) biplot illustrating the relationships among the biochemical composition of bitter orange (*Citrus aurantium*), grapefruit (*Citrus paradisi*), sweet orange (*Citrus sinensis*), and mandarin (*Citrus reticulata*). The first principal components (PC1) and second principal components (PC2) accounted for 86.3 percent of the total variance of the changes. The PC1 accounting for 68.4 percent (eigen value=5.47), and the PC2 accounting for 17.9 percent (eigen value=1.43). The PC1 is positively correlated with sweet orange and mandarin and energy (0.343), carbohydrate (0.312), fat (0.283), and dry matter (0.400). PC1 is negatively correlated with sweet orange and mandarin and protein (-0.411), digestibility (-0.403), ash (-0.232), and moisture (-0.400). The PC2 is positively correlated with sweet orange and bitter orange and dry ash (0.518), fat (0.511), energy (0.211), matter (0.193), and digestibility (0.162). PC2 is negatively correlated with mandarin, grapefruit, and carbohydrate (-0.571), and moisture (-0.193).


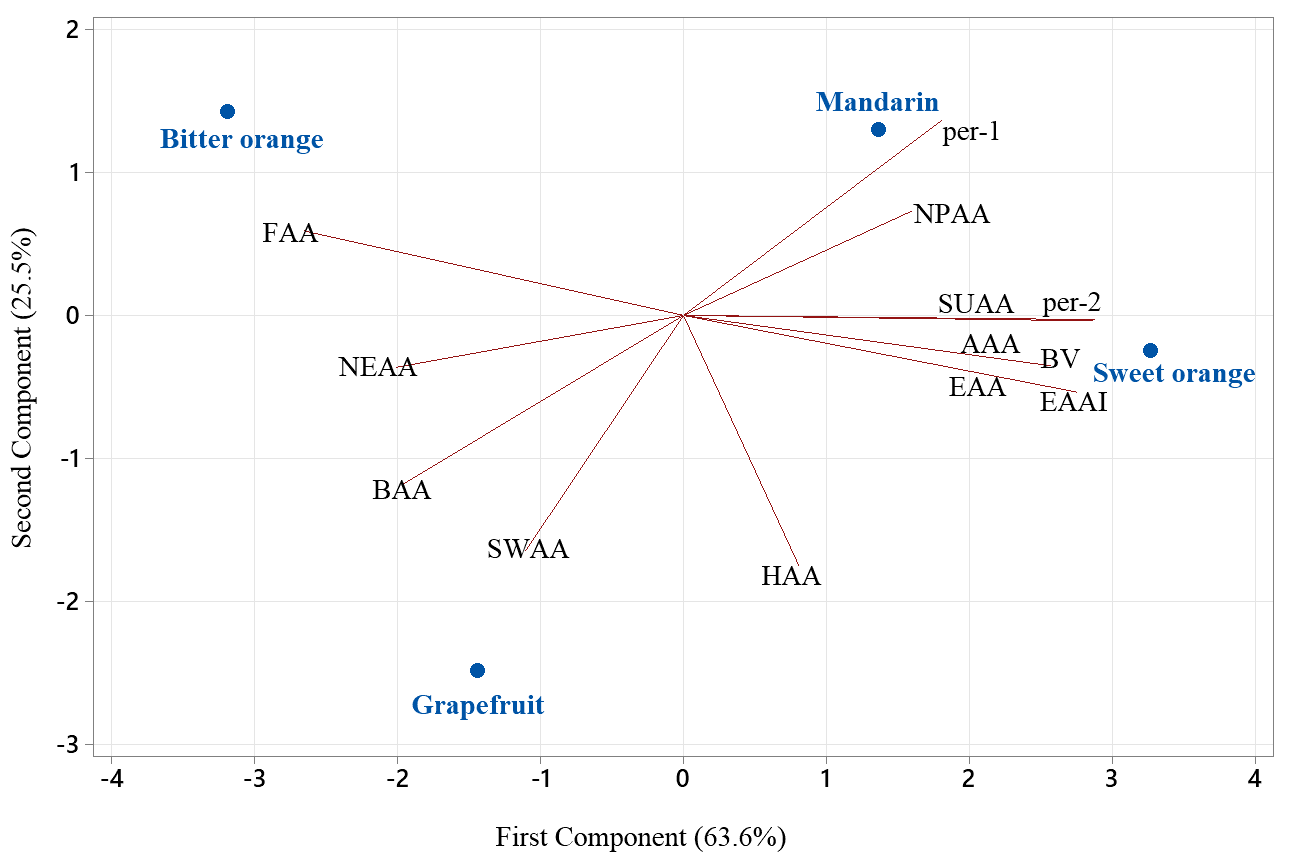


Figure S2. Principal Component Analysis (PCA) biplot illustrating the relationships among the amino acid nutritional quality of bitter orange (*Citrus aurantium*), grapefruit (*Citrus paradisi*), sweet orange (*Citrus sinensis*), and mandarin (*Citrus reticulata*). The PC1 and PC2 accounted for 89.1 percent of the total variance of the changes in nutritional quality. The PC1 accounting for 63.6 percent (eigen value=8.26) and the PC2 accounting for 25.5 percent (eigen value=3.31). The PC1 is positively correlated with sweet orange and mandarin and PER2 (0.348), SUAA (0.346), EAAI (0.332), BV (0.332) EAA (0.332), AAA (0.311), PER1 (0.218), and NPAA (0.193) variable. PC1 is negatively correlated with grapefruit and bitter orange and FAA (-0.321), NEAA (-0.243), BAA (-0.238), and SWAA (-0.133) variables. The PC2 is positively correlated with bitter orange and mandarin and PER1 (0.411), NPAA (0.220), and FAA (0.178) and variables. PC2 is negatively correlated with grapefruit and sweet orange and HAA (-0.527), SWAA (-0.495), BAA (-0.356), EAA (-0.161), EAAI (-0.161), and BV (-0.161), NEAA (-0.109), and AAA (-0.106).


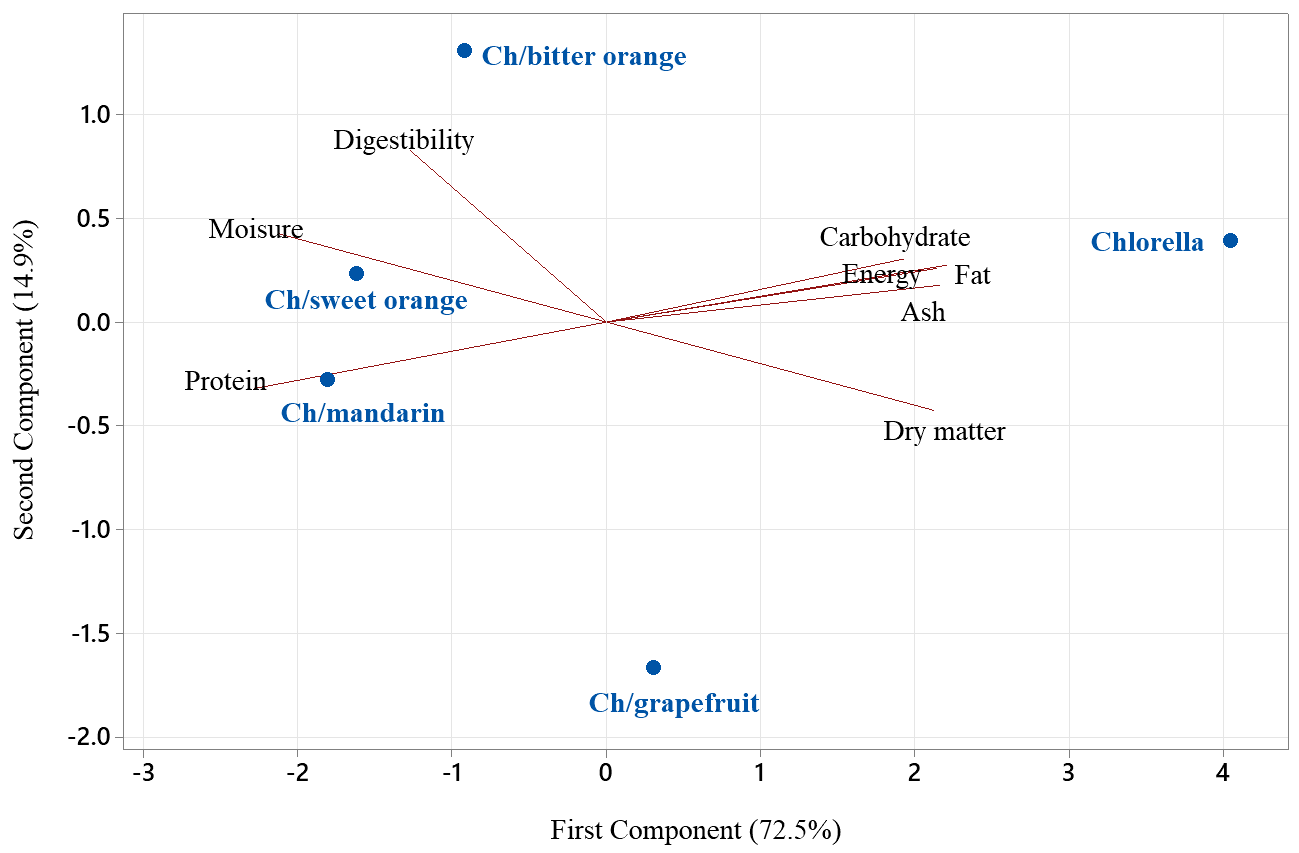


Figure S3. Principal Component Analysis (PCA) biplot illustrating the relationships among the biochemical composition of *Chlorella vulgaris* (Ch) supplemented with bitter orange (*Citrus aurantium*), grapefruit (*Citrus paradisi*), sweet orange (*Citrus sinensis*), and mandarin (*Citrus reticulata*). The PC1 and PC2 accounted for 87.4 percent of the overall variance of the changes. PC1 accounts for 72.5 percent (eigen value=5.79), whereas PC2 accounts for 14.9 percent (eigen value=1.19) of the total variance. The PC1 is positively correlated with chlorella and *Chlorella/*grapefruit and is closely associated with fat (0.381), ash (0.372), energy (0.369), dry matter (0.365), and carbohydrate (0.333). PC1 is negatively correlated with *Chlorella/*bitter orange, *Chlorella/*mandarin, and *Chlorella/*sweet orange and with protein (-0.394), moisture (-0.365), and digestibility (-0.220). The PC2 is positively correlated with *Chlorella/*bitter orange, and *Chlorella/*sweet orange and digestibility (0.697), moisture (0.357), carbohydrate (0.256), fat (0.231), energy (0.217), and ash (0.149). PC2 negatively correlates with *Chlorella/*mandarin, *Chlorella/*grapefruit, dry matter (-0.357), and protein (-0.269).


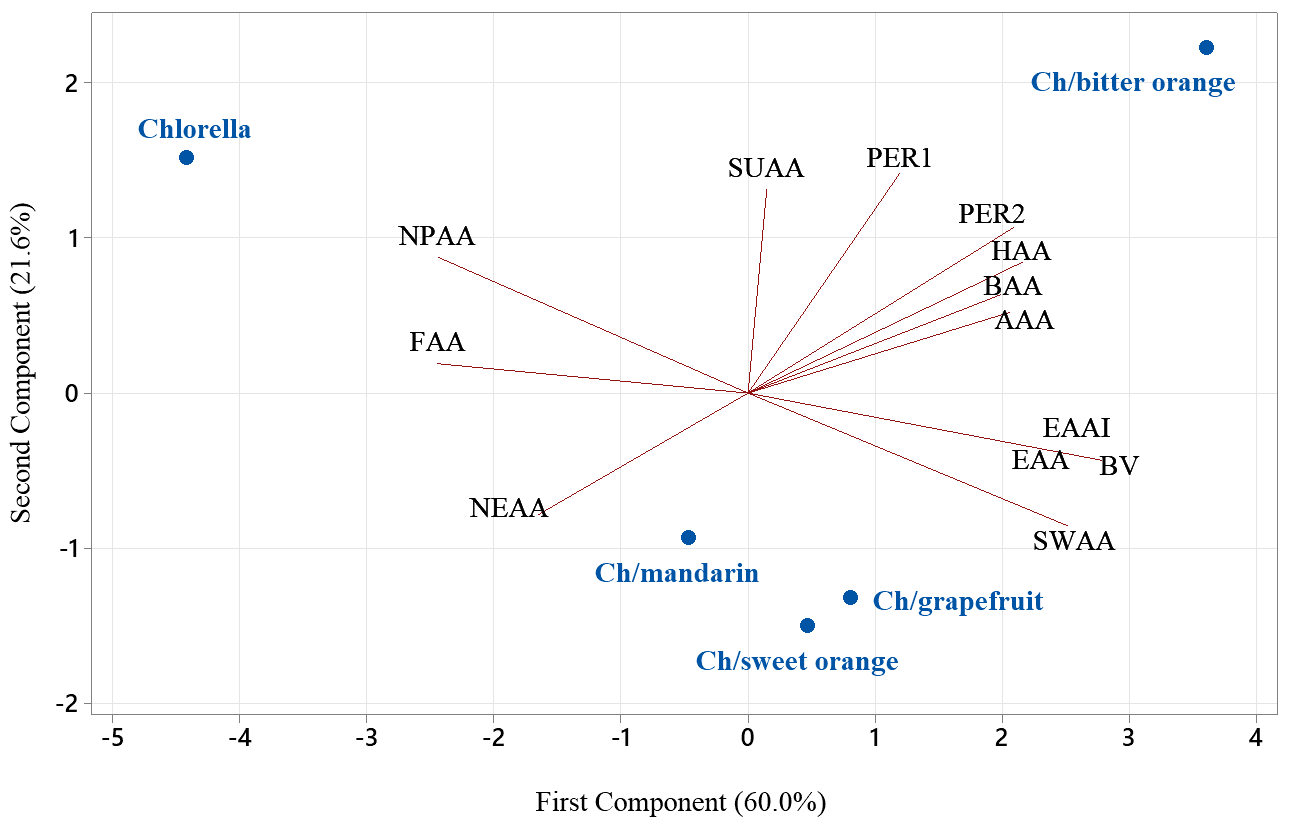


Figure S4. Principal Component Analysis (PCA) biplot illustrating the relationships among the amino acid nutritional quality of *Chlorella vulgaris* supplemented with bitter orange (*Citrus aurantium*), grapefruit (*Citrus paradisi*), sweet orange (*Citrus sinensis*), and mandarin (*Citrus reticulata*). The PC1 and PC2 accounted for 81.6 percent of the overall variance changes. The PC1 is responsible for 60.0 percent (eigen value=8.39), while the PC2 is responsible for 12.6 percent (eigen value=3.02). The PC1 is positively correlated with *Chlorella/*bitter orange, *Chlorella/*grapefruit, and *Chlorella*/sweet orange, and EAA (0.331), EAAI (0.331), BV (0.331), SWAA (0.299), HAA (0.257), PER2 (0.249), AAA (0.245), BAA (0.237), and PER1 (0.142). PC1 is negatively correlated with *Chlorella* and *Chlorella*/mandarin and FAA (-0.291), NPAA (-0.290), and NEAA (-0.197). The PC2 is positively correlated with *Chlorella* and *Chlorella/*bitter orange samples and PER1 (0.469), PER2 (0.352), SUAA (0.434), NPAA (0.289), HAA (0.279), BAA (0.209), and AAA (0.172). PC2 is negatively correlated with *Chlorella/*grapefruit, *Chlorella/*sweet orange, and *Chlorella/*mandarin and SWAA (-0.282), NEAA (-0.259), EAA (-0.143), EAAI (-0.143), and BV (-0.143).


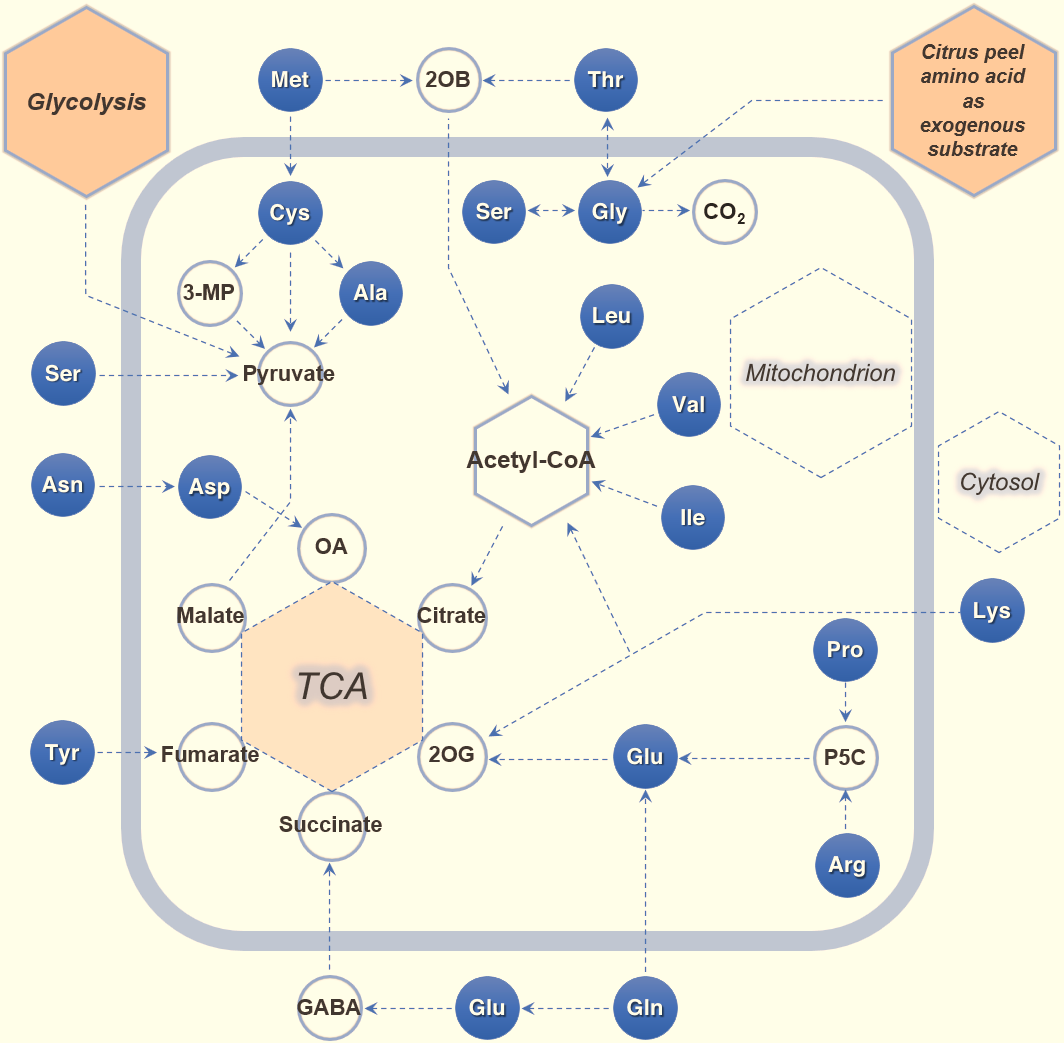


Figure S5**.** Amino acid catabolism in the presence of citrus peels amino acid (adapted from Hildebrandt et al., 2015; Keaton et al., 2013). Amino acids (blue circles) are broken down into precursors or mediators of the TCA cycle, most of which occur in the mitochondria. Citrus peel amino acids can enter the catabolism pathway of *Chlorella vulgaris* through the glycine or any other amino acid. Abbreviations: 2OB, 2-oxobutyrate; 2OG, 2-oxoglutarate; OA, oxaloacetate; GABA, γ-aminobutyric acid; P5C, 1-pyrroline-5-carboxylate; 3MP, 3-mercaptopyruvate. TCA=tricarboxylic acid; Met=Methionine; Glu=Glutamic acid; Gln=Glutamine; Arg=Arginine; Pro=Proline; Lys=Lysine; Tyr= Tyrosine; Asn=Asparagine; Asp=Aspartic acid; Ser=Serine; Ala=Alanine; Cys=Cysteine; Gly=Glycine; Val=Valine; Leu=Leucine; Ile=Isoleucine; Thr=Threonine.


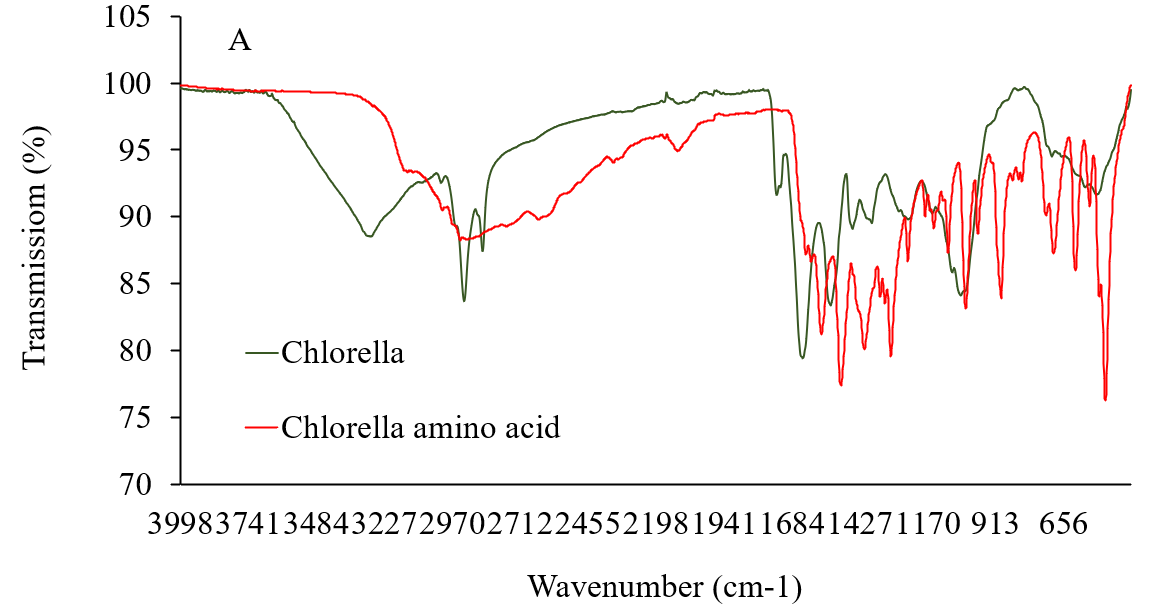


Figure S6. Fourier transform the infrared spectrum of *Chlorella* and *Chlorella* amino acid.


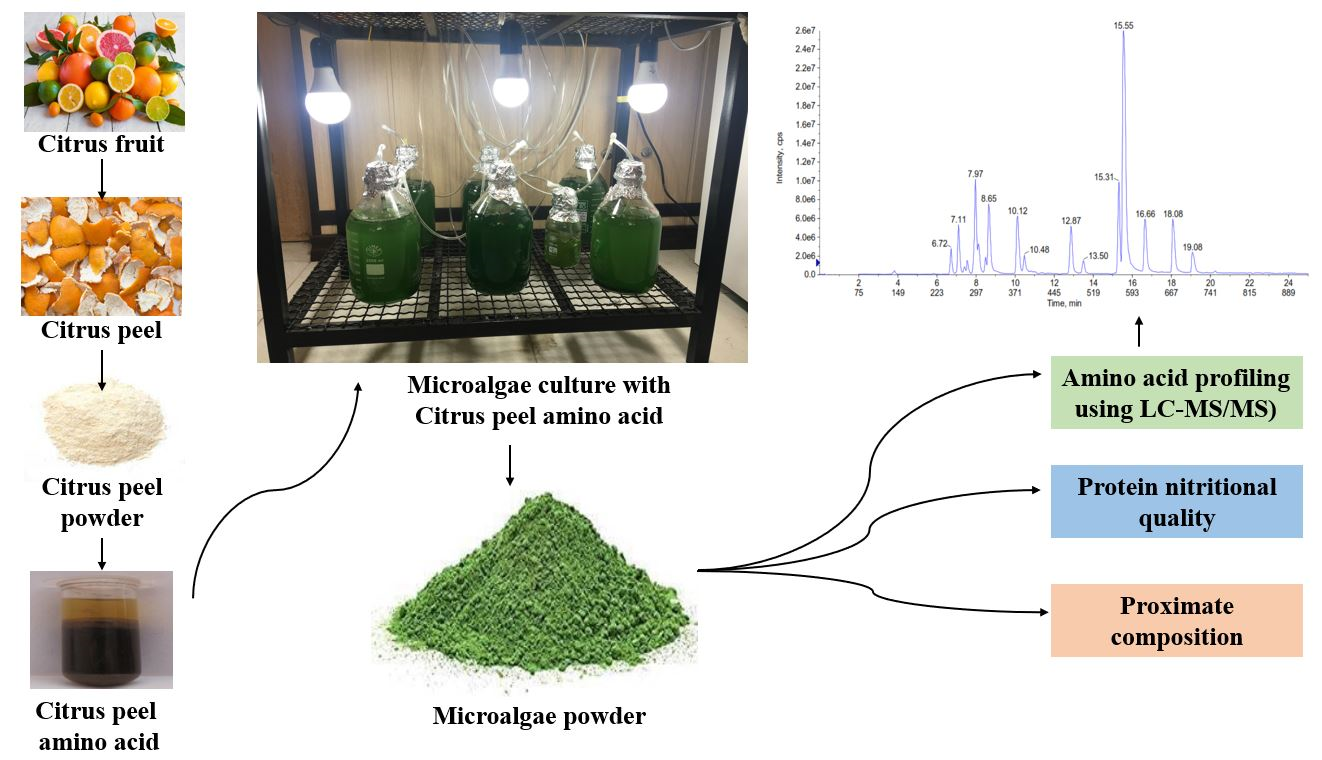


Figure S7. Graphical abstract.
